# Cutaneous Melanoma and Occupational UV Exposure: Associations with Anatomical Site, Histological Subtype, and Breslow Thickness

**DOI:** 10.3390/cancers17162705

**Published:** 2025-08-20

**Authors:** Vincenzo De Giorgi, Silvia Viscera, Giovanni Cecchi, Elisabetta Magnaterra, Veronica Traversini, Gabriella Perillo, Biancamaria Zuccaro, Federica Fazzari, Antonio Baldassarre, Stefano Dugheri, Nicola Mucci

**Affiliations:** 1 Department of Health Sciences, Section of Dermatology, University of Florence, 50125 Florence, Italy; giovanni.cecchi1@unifi.it (G.C.); elisabetta.magnaterra@unibo.it (E.M.); gabriella.perillo@unifi.it (G.P.); biancamaria.zuccaro@unifi.it (B.Z.); federica.fazzari@unifi.it (F.F.); 2 Cancer Research ‘Attilia Pofferi’ Foundation, 51100 Pistoia, Italy; 3 Department of Experimental and Clinical Medicine, University of Florence, 50134 Florence, Italy; silvia.viscera@unifi.it (S.V.); veronica.traversini@unifi.it (V.T.); antonio.baldassarre@unifi.it (A.B.); nicola.mucci@unifi.it (N.M.); 4 Department of Life Science, Health, and Health Professions, Link Campus University, 00165 Rome, Italy; s.dugheri@unilink.it

**Keywords:** lentigo maligna, skin cancer, sun exposure, outdoor workers

## Abstract

**Background:** The relationship between occupational sun exposure and melanoma risk is complex and multifaceted, with existing evidence yielding contradictory findings. Unlike Non-Melanoma Skin Cancer (NMSC), for which occupational sun exposure is a well-established risk factor, the link with cutaneous melanoma remains contentious. **Objectives:** This study aimed to evaluate whether, in a cohort of patients with cutaneous melanoma, an association existed between occupational sun exposure and melanoma, specifically with histotype, site of occurrence, and Breslow index. **Methods:** This is a retrospective cohort analysis conducted to evaluate whether occupational sun exposure constitutes a risk factor for the development of cutaneous melanoma in patients diagnosed between January 2005 and October 2023 at the Dermatology Unit, Azienda USL Toscana Centro, Florence. Occupational ultraviolet (UV) exposure was examined by classifying each participant’s job into categories based on solar UV exposure levels—outdoor (e.g., agriculture and construction roles), mixed indoor/outdoor (e.g., trades and public safety professions), and indoor settings (e.g., office-based work). **Results:** A final total of 1417 patients were analyzed. Occupational categorization revealed that 1171 patients (82.64%) were classified as non-occupationally exposed (indoor), while 246 (17.36%) were occupationally exposed to solar UV radiation (including 14.82% mixed indoor/outdoor and 2.54% outdoor workers). A significant association was observed between occupational sun exposure and lentigo maligna, which was more prevalent among exposed workers and even more so in the outdoor subgroup. Anatomical site distribution exhibited a significant association with occupational sun exposure. Indeed occupationally exposed individuals showed a higher prevalence of melanomas in the head and neck region, a distribution pattern particularly evident among outdoor workers, suggesting that these sites may be more susceptible to chronic sun exposure in outdoor and mixed occupations. Moreover, a significant association was found between occupational exposure and Breslow thickness, with exposed workers presenting with thicker melanomas at diagnosis, suggesting more advanced disease. **Conclusions:** The finding of this study may reflect variations in occupational sun exposure patterns and warrants further investigation into protective measures and early-detection strategies tailored to occupational groups.

## Simple Summary

Exposure to sunlight at work is common for many people, especially those with outdoor jobs such as construction workers, farmers, and lifeguards. While it is well known that sunlight can cause certain skin cancers, its role in melanoma remains unclear. In this study, we looked at over 1400 patients diagnosed with melanoma in Italy to understand if people exposed to the sun through their job are at higher risk. We compared patients’ job types, the location of the melanoma on their bodies, and the type and depth of the tumors. Our findings suggest that sun exposure at work may affect where melanoma appears and how advanced it is at diagnosis. This research may help doctors and public health experts improve skin cancer prevention strategies, especially for workers exposed to the sun regularly.

## 1. Introduction

Sun exposure (SE) is the predominant source of UV radiation exposure for humans and presents a critical occupational health risk. The Carcinogen Exposure (CAREX) database highlights SE as a significant contributor to occupational carcinogenic risk, an issue of increasing concern [1,2]. In Europe alone, approximately 15 million outdoor workers (OW) experience substantial exposure to UV-A and UV-B radiation, encompassing up to 75% of their work hours, as reported by the European Agency for Safety and Health at Work (EU-OSHA) [3]. Despite this extensive exposure, a notable regulatory gap persists due to the exclusion of SE from the European Directive 2006/25/EC, creating a void in preventive measures and resulting in the absence of exposure limit values (ELVs) specific to solar radiation [4].

Numerous studies worldwide have quantified UV radiation exposure among outdoor workers, consistently demonstrating exposure levels that exceed current occupational thresholds established for artificial UV radiation by bodies such as the American Conference of Governmental Industrial Hygienists (ACGIH) and the International Commission on Non-Ionizing Radiation Protection (ICNIRP). For instance, considering the occupational exposure limit of 30 Joules per square meter (equivalent to 1–1.3 Standard Erythema Doses for fair skin), exposure levels for outdoor workers often surpass these limits more than tenfold [5].

A systematic review examining the association between occupational roles and melanoma incidence revealed mixed outcomes across 65 studies, with sectors such as agriculture, air transport, electrical, healthcare, research, nuclear, oil, tanning, pulp, and education reporting varied results. Of ten studies specifically exploring the link between occupational UV exposure and melanoma, five found a positive association among outdoor workers, whereas two noted a positive association among indoor occupations [6].

Studies further exploring melanoma localization have identified a potential association between occupational SE and melanomas of the head and neck [7]. However, other research contradicts this finding, suggesting no significant or even a negative association between occupational SE and cutaneous melanoma, including head and neck melanomas [8,9,10]. A plausible explanation for this paradox is that continuous SE induces skin thickening, photoadaptation, and increased melanin production, offering protective and antioxidant properties that may mitigate melanoma risk [8].

While outdoor workers exhibit a heightened risk of NMSC, such as squamous cell carcinoma and basal cell carcinoma, the relationship of outdoor work with melanoma remains complex [8]. A large prospective cohort study spanning cancer registries from Denmark, Finland, Norway, Sweden, and Australia examined melanoma risk based on occupational history, categorizing workers as indoor, outdoor, or mixed indoor/outdoor. It found a higher standardized incidence ratio (SIR) of cutaneous melanoma among indoor workers (SIR 1.09) compared to outdoor workers (men: SIR 0.79; women: SIR 0.92). However, this finding should not be misinterpreted as evidence of protective effects from outdoor work. Reduced risk among outdoor workers may reflect lower UV exposure at higher latitudes or the use of protective clothing in colder climates [11].

Global recognition of cutaneous tumors as occupational diseases remains inconsistent. A comparative legislative analysis across 11 European countries revealed that squamous cell carcinoma is acknowledged as occupational in seven countries, basal cell carcinoma in six, actinic keratosis in five, and Bowen’s disease in five. Malignant melanoma is recognized as occupational in only three countries (Denmark, Romania, and Portugal), whereas the United Kingdom, Croatia, Sweden, and Poland do not formally classify any type of skin cancer as potentially occupational in origin [2]. These disparities underscore the need for harmonized policies addressing occupational SE and its carcinogenic implications.

In our study, we aimed to evaluate, in a group of patients with cutaneous melanoma, whether there was an association between occupational SE and melanoma specifically with regard to the histotype, site of occurrence, and Breslow index.

## 2. Methods

The present study is a retrospective cohort analysis conducted to evaluate whether occupational SE constitutes a risk factor for the development of cutaneous melanoma in patients diagnosed between January 2005 and October 2023 at the Dermatology Unit, Azienda USL Toscana Centro, Florence. The study included individuals with confirmed diagnoses of cutaneous melanoma and examined a range of clinical, demographic, and lifestyle variables to assess potential associations with melanoma risk. The primary variables of interest encompassed demographic, clinical, and lifestyle factors, including gender, phototype, age at first melanoma diagnosis, the number of common naevi, educational level, family history of melanoma, anatomical melanoma location, Body Mass Index (BMI), recreational sun exposure, sunburn frequency and age at first sunburn, latency between initial sunburn and melanoma onset, prior radiotherapy, use of artificial UV tanning devices, smoking status, and presence of metastasis at diagnosis. Occupational UV exposure was examined by classifying each participant’s job into categories based on solar UV exposure levels—outdoor (e.g., agriculture and construction roles), mixed indoor/outdoor (e.g., trades and public safety professions), and indoor settings (e.g., office-based work). Notably, air pilots and flight attendants, despite primarily indoor work conditions, were classified as mixed exposure due to potential UV penetration through windows. This categorization followed established classifications [8,11,12], but lacked quantitative UV exposure measures such as UV index and seasonal variations. Information regarding the use of sun protection measures, including sunscreen, was not captured within the dataset. Full occupational classifications are provided in Table 1. For histopathological characteristics, each case was reviewed for the histological diagnosis and Breslow thickness of melanoma at initial presentation.

The retrospective nature of this study imposes certain inherent limitations upon it. The occupational classification was based on literature-derived categories without specific UV risk measurements for individual cases. Additionally, certain confounding factors could not be fully addressed, such as sun exposure-related variables, including sunscreen use, melanoma mortality rates, and the presence of NMSC (such as squamous and basal cell carcinomas) associated with chronic UV exposure. Data were collected from the Dermatology Unit database, which provided partial information on patients’ occupational histories and lifestyle factors related to sun exposure. Descriptive statistics were first applied to summarize the distribution of key demographic and clinical characteristics. Frequency tables were generated for univariate and bivariate distributions, and contingency tables were used for stratified analyses involving three variables where appropriate. The Chi-square test for independence (χ^2^ test) was used to assess associations between categorical variables, with the results reported as observed test statistics and corresponding *p*-values. A two-tailed significance threshold of α = 0.05 was adopted. Where applicable, odds ratios (ORs) and 95% confidence intervals (CIs) were calculated to estimate the strength and precision of associations. All statistical analyses were conducted using Stata version 17 (StataCorp LLC, College Station, TX, USA) and Jamovi version 2.4 (The Jamovi Project).

## 3. Results

Our study initially included a cohort of 1928 patients. After applying exclusion criteria, a final total of 1417 patients were analyzed. Exclusions were based on the following criteria: patients with unknown occupational data, retirees with untraceable prior occupations, unemployed individuals, homemakers, minors under 18 years old (as the study focused on workers), patients with unclassifiable or unknown histological diagnoses, and cases of genital mucosal melanoma due to its distinct etiopathogenesis.

The analyzed cohort comprised 779 male (55%) and 638 female (45%) patients. The majority (65.9%) had fair phototypes (Fitzpatrick I and II), aligning with established data indicating increased melanoma prevalence in fair-skinned populations. Occupational categorization revealed that 1171 patients (82.64%) were classified as non-occupationally exposed (indoor), while 246 (17.36%) were occupationally exposed to solar UV radiation (including 14.82% mixed indoor/outdoor and 2.54% outdoor workers).

**Figure 1:**
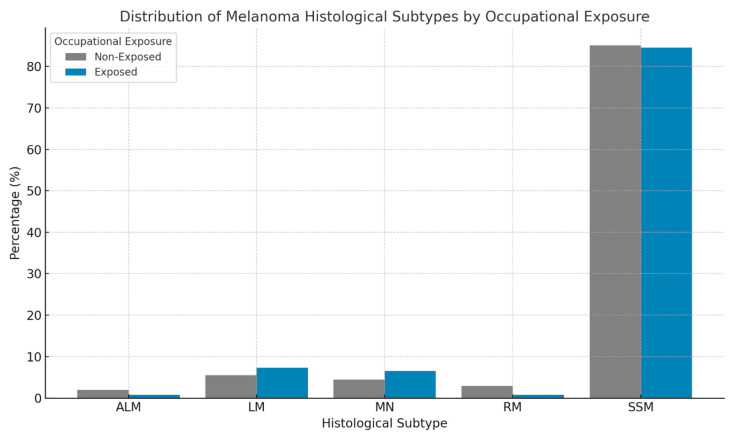
Percentage distribution of melanoma histological subtypes by occupational exposure. Superficial spreading melanoma (SSM) was the most frequent histological subtype in both groups, representing the majority of cases among indoor workers (997 cases) as well as among those with occupational exposure (208 cases, including mixed and outdoor workers). Lentigo maligna, typically associated with chronic sun damage, showed a relatively higher proportion in occupationally exposed individuals (18 cases), especially among outdoor workers, compared to indoor workers (65 cases). Nodular melanoma and acral lentiginous melanoma were less common overall and showed a more uniform distribution across the categories.

The histological subtype most frequently observed was superficial spreading melanoma (SSM), accounting for 85.04% of cases, followed by lentigo maligna (5.86%), nodular melanoma (4.80%), rare histological subtypes (2.54%), and acral lentiginous melanoma (1.76%) (Figure 1). Subtype prevalence was consistent across occupational categories, with superficial spreading melanoma comprising the majority in the indoor (85.14%), mixed (85.71%), and outdoor (77.78%) worker groups.

Conversely, lentigo maligna was significantly more prevalent among outdoor workers (13.89%) compared to the indoor (5.55%) and mixed (6.19%) categories, with a statistically significant difference (OR: 2.69; 95% CI: 1.02–7.12; *p* = 0.046). No significant differences were found between occupational categories regarding nodular, acral lentiginous, or rare melanoma subtypes.

**Figure 2:**
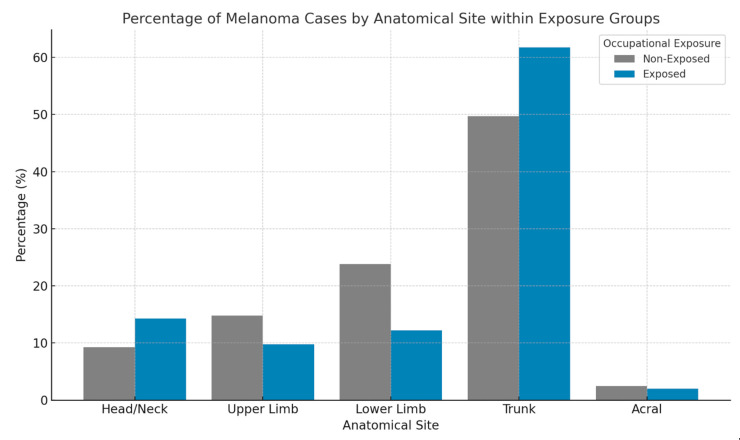
Percentage distribution of melanoma cases by anatomical site within occupational exposure groups. In indoor workers, melanomas most frequently affected the trunk (581 cases) and lower limbs (278 cases), with fewer cases on the head and neck (108), upper limbs (174), and acral regions (29). Among occupationally exposed individuals (mixed + outdoor), the trunk remained the most commonly affected site (134 cases), followed by the head and neck (35 cases), lower limbs (30 cases), upper limbs (24 cases), and acral areas (5 cases). These patterns suggest a relative increase in head and neck involvement among exposed individuals, consistent with chronic sun exposure.

Moreover, anatomical site distribution did exhibit a significant association with occupational solar exposure. Among non-occupationally exposed patients (indoor workers), melanomas predominantly affected the trunk (49.7%), followed by the lower limbs (23.78%), upper limbs (14.8%), head and neck region (9.24%), and acral areas (2.48%). In contrast, occupationally exposed patients (outdoor and mixed categories) presented with a higher prevalence of trunk melanomas (61.79%) and head and neck melanomas (14.23%), with a lower prevalence of lower limb (12.2%), upper limb (9.76%), and acral (2.03%) melanomas (Figure 2). These findings indicate a significantly higher incidence of melanomas on the limbs among indoor workers (OR = 2.227; 95% CI: 1.611–3.079; *p* < 0.001), whereas occupationally exposed individuals had higher rates of trunk and especially head and neck melanomas (OR = 1.630; 95% CI: 1.083–2.453; *p* = 0.018). Within the head and neck region, the risk further increased for outdoor workers (OR = 2.633; 95% CI: 1.176–5.892; *p* = 0.015).

Further analysis revealed a significant association between occupational solar exposure and Breslow thickness (OR = 1.509; 95% CI: 1.083–2.103; *p* = 0.015). Non-occupationally exposed patients demonstrated a higher proportion of melanomas with a Breslow thickness ≤ 1 mm (83%) compared to occupationally exposed patients (76%). It should be noted that the odds ratios (ORs) reported in this section refer to associations within this patient cohort and do not represent incidence rates or standardized comparisons across populations (e.g., SIRs).

## 4. Discussion

The relationship between occupational SE and melanoma risk is complex and multifaceted, with existing evidence yielding contradictory findings. Unlike NMSC, for which occupational SE is a well-established risk factor, the link with cutaneous melanoma remains contentious. Some studies have demonstrated a positive association between outdoor work and melanoma risk, while others have either reported a higher risk for indoor workers or found no association at all [8,11,12,13]. In our cohort, 83% of patients were classified as non-occupationally exposed (indoor workers). While this distribution might suggest a predominance of melanoma diagnoses among indoor workers, it should not be interpreted as an indication of higher melanoma risk in this group. Since this is a hospital-based cohort and not a population-based sample, no inference on incidence rates can be made. Moreover, although it is plausible that this proportion reflects the general occupational distribution in the Florence area, region-specific comparative data are not available to confirm this. Surveillance bias may also contribute to this finding, as indoor workers—often associated with higher socioeconomic status—may have more frequent access to dermatological screening and earlier diagnosis. Additionally, lifestyle-related factors such as intermittent, intense sun exposure during holidays or tanning bed use may influence melanoma development in indoor workers.

Superficial spreading melanoma (SSM) was confirmed as the most frequent histological subtype across both the occupationally exposed and non-exposed groups. However, a significant association was observed between occupational SE and lentigo maligna, which was more prevalent among exposed workers and even more so in the outdoor subgroup.

This finding was statistically significant and aligns with the established pathogenesis of lentigo maligna, a subtype that typically arises in chronically sun-damaged skin and is histologically marked by pronounced solar elastosis [14]. These findings reinforce the hypothesis that long-term cumulative UV exposure, especially in occupational contexts, may substantially contribute to the pathogenesis and clinical development of this melanoma subtype.

Anatomical site distribution exhibited a significant association with occupational sun exposure. Among indoor workers, a significant association was observed with limb melanomas, likely reflecting differences in UV exposure and protective behaviors. In contrast, occupationally exposed individuals showed a higher prevalence of melanomas in the head and neck region, a distribution pattern particularly evident among outdoor workers, suggesting that these sites may be more susceptible to chronic SE in outdoor and mixed occupations. This finding aligns with some studies that have reported an elevated risk of head and neck melanomas in outdoor workers due to chronic UV exposure [7,14].

Additionally, a significant association was found between occupational exposure and Breslow thickness, with exposed workers presenting with thicker melanomas at diagnosis, suggesting more advanced disease. This may reflect a diagnostic delay among outdoor and mixed-exposure workers, potentially due to a combination of factors. These include lower socioeconomic status and health literacy, which can reduce engagement in preventive care, as well as practical barriers such as inflexible work schedules or limited access to dermatologic services. Moreover, melanoma lesions arising on chronically sun-damaged skin—common in outdoor workers—may be more difficult to recognize, either by patients or clinicians, leading to further delays. These considerations underscore the need for targeted public health interventions aimed at promoting earlier detection and facilitating access to dermatologic care in occupationally vulnerable populations.

In this context, prevention strategies should extend beyond simple sun avoidance to incorporate tailored educational and awareness campaigns targeting both indoor and outdoor workers. Occupational health guidelines must consider not only regulatory compliance but also the practical realities of worker exposure. Preventive measures should include shade structures, modified work hours to reduce peak UV exposure, personal protective equipment, and the use of high-SPF sunscreens. Education on the risks of occupational sun exposure, along with information on photosensitizing agents that interact with UV radiation, remains a key pillar of prevention.

Moreover, our findings that occupational SE influences melanoma anatomical distribution and, to some extent, disease severity at diagnosis reinforce the importance of targeted surveillance. Tailoring prevention efforts based on occupational exposure patterns can facilitate more effective screening, potentially improving outcomes for high-risk groups.

Understanding and addressing the role of occupational SE within the broader context of melanoma risk is therefore vital. This requires not only legislative support and workplace compliance but also comprehensive, proactive strategies that empower workers to protect themselves against UV radiation, reduce melanoma risk, and improve overall public health outcomes.

Nevertheless, a key limitation of this study lies in the qualitative nature of the sun exposure assessment, which was based solely on occupational classification rather than on direct or individualized measurements. While this approach is widely used in epidemiological research, it may lead to exposure misclassification. For instance, indoor workers may experience substantial recreational UV exposure, while outdoor workers might adopt strict photoprotection practices or work in shaded environments, resulting in lower actual exposure than expected.

In particular, the “mixed” exposure category introduces an additional layer of complexity, as it includes a heterogeneous set of occupations with potentially diverse UV exposure profiles. For example, truck drivers, tour guides, and real estate agents may all fall into this group, despite notable differences in their actual time spent under direct sunlight. This heterogeneity may have led to differential misclassification within the category, potentially attenuating or obscuring associations.

The lack of detailed data on exposure intensity, duration, seasonal variation, and the use of sun protection limits the precision of our estimates and the generalizability of our findings. Future studies should aim to incorporate more granular, quantitative exposure data—such as self-reported UV exposure histories or job-exposure matrices—to better capture individual variability and improve risk estimation accuracy.

## 5. Conclusions

This study demonstrates that occupational sun exposure significantly influences the anatomical distribution, histological subtype, and Breslow thickness of cutaneous melanoma. In particular, outdoor and mixed-exposure workers showed a higher prevalence of head and neck melanomas and the lentigo maligna subtype, and thicker tumors at diagnosis. These findings suggest that cumulative UV exposure in occupational settings plays a relevant role in melanoma development and severity. Recognizing this association is crucial for designing targeted preventive measures, including early-detection strategies and occupational health policies that address UV-related risks. A stronger focus on public health education, protective practices, and regulatory harmonization across countries may help reduce the burden of occupation-related melanoma.

## Figures and Tables

**Table 1:** Classification of occupational activities based on solar UV exposure.

Potential Occupational Solar UV Exposure	Occupation
**Outdoor**	Sailors, farmers, agricultural workers, fishers, gardeners, construction workers (bricklayers, crane operators, carpenters), lifeguards, postal workers/couriers.
**Mixed Indoor/Outdoor**	Waste collectors, mechanics, carpenters, electricians, plumbers, painters, chimney sweeps, blacksmiths, public safety workers (law enforcement, firefighters), military personnel, caretakers/custodians, transport sector workers (drivers, pilots, lorry drivers), real estate agents, tour guides, kindergarten educators, warehouse staff, maintenance workers, technicians (engineers, surveyors, geologists), street vendors, event organizers.
**Indoor**	All others: teachers, professors, office workers, healthcare professionals, shopkeepers, etc.

## Data Availability

The data that support the findings of this study are available from the corresponding author upon reasonable request.
